# Immunoassay of Glomalin by Quartz Crystal Microbalance Biosensor Containing Iron Oxide Nanoparticles

**DOI:** 10.1155/2020/8844151

**Published:** 2020-09-01

**Authors:** Miroslav Pohanka, Vitezslav Vlcek

**Affiliations:** ^1^Faculty of Military Health Sciences, University of Defence, Trebesska 1575, CZ-500 01, Hradec Kralove, Czech Republic; ^2^Faculty of AgriSciences, Mendel University in Brno, Zemedelska 1, 613 00, Brno, Czech Republic

## Abstract

Glomalin is a soil protein resembling heat shock protein (HSP) 60 and exerting high affinity to metals, causing retention of water in the environment and improving mechanical stability of soil. Currently, glomalin is determined in the soil or other samples by combination of autoclaving extraction and total protein determination typically by the Bradford method. In this paper, a piezoelectric biosensor was prepared to determine glomalin in a label-free measurement. The biosensor contained antibodies immobilized on quartz crystal microbalance (QCM), and the recognition layer was stabilized by iron oxide nanoparticles. The assay was tested on real soil samples and compared with the standard Bradford assay. Limit of detection of the assay was equal to 2.4 *µ*g/g for a soil extract with a volume of 50 *µ*l. The assay takes approximately half of an hour and was fully correlated to the Bradford assay. The biosensor had significant advantages than the other methods: it worked in a label-free mode and was fully applicable for practical samples.

## 1. Introduction

Presence of free proteins in the environment has been underestimated for a long time. Assay of this proteins can help in various processes and may be helpful, for instance, in the criminalistics, archeology, agronomy, ecology, and other disciplines. Glomalin is one of the proteins that can be found in the nature in an excessive amount. In the current literature, glomalin is frequently called as glomalin-related soil proteins because the information about it is quite insufficient and it is not easy to distinguish between them. In its structure, glomalin exerts high homologies with health shock protein 60 (HSP 60) including the human type, but the soil glomalin is of a fungal origin and it is a product of arbuscular mycorrhizal fungi [1–3]. Currently, glomalin is extensively studied, and its presence in the nature appears to have a significant role for water and metals retaining mechanical stability of soil, ability of microorganism surviving in the environment, and so on. It exerts high stability which is in a good correspondence with knowledge about HSP 60.

Standard determination of glomalin-respective glomalin-related proteins is based on heat extraction by autoclaving to citrate buffer followed by total protein measurement by, e.g., Bradford assay [4–9]. The aforementioned protocol is used in this paper as a standard method, and it is described in the experimental part. Methods based on specific antibodies like enzyme-linked immunosorbent assay (ELISA) are another way to measure glomalin concentration in natural samples [4, 10]. Other types of immunoassays like photothermal immunoassays, point-of-care immunoassays, photoelectrochemical immunoassays, and colorimetric immunoassays can serve as the platform in the future as well [11–16]. The immunoassays can be considered as more accurate though the extraction, and total protein determination is an assay of the first choice in the current praxis.

This paper is devoted to the construction of a biosensor for the glomalin assay. Piezoelectric quartz crystal microbalance (QCM) sensor was chosen as a platform for the biosensor construction, and iron oxide nanoparticles were added to the recognition antibody as an improvement for detected signal. The QCM sensors are recording mass bound on their surface, and improving of the surface by an antibody immobilization makes them an ideal tool for a fast and easy to process analysis of various chemical structures. The drift of oscillation frequency due to chemically attached material on the QCM surface is the physical principle of the assay [17, 18]. Working in the label-free mode, cheap equipment for the assay, and availability of QCM because they are mass produced for electronic industry are the significant advantages and reasons why the QCM-based biosensors have practical sense [17–21]. It is expected that the biosensor will be an applicable tool for glomalin assay and will be fully competitive to the standard protocols.

## 2. Materials and Methods

### 2.1. Preparation of Activated Iron Oxide Nanoparticles

Iron oxide (II, III) nanoparticles with diameter 30 nm, covered with carboxylic acid, and magnetization ˃45 emu/g (at room temperature; under 4500 Oe) were purchased from Sigma-Aldrich (Sigma-Aldrich; St. Louis, MO, USA). The particles were washed by phosphate-buffered saline pH 7.4 (PBS); a permanent magnet was used for separation of the nanoparticles between each step. After washing, *N*-(3-dimethylaminopropyl)-*N*′-ethylcarbodiimide hydrochloride (Litolab, Chudobin, Czech Republic) 20 mg/ml in PBS was used for suspension with the nanoparticles making. The suspension was let to incubate for half of an hour, and then, the nanoparticles were washed by PBS again.

### 2.2. Immobilization of Antibody on QCM

The biosensors were prepared on the basis of 10 MHz QCM sensors (manufacturer Krystaly, Hradec Kralove, Czech Republic). The sensor had following specifications: a diameter of quartz disc 20 mm, diameter of two gold electrodes on the opposite surfaces of quartz disc 7 mm, the gold material was deposited on chromium interlayer, and thickness of quartz disc 0.166 mm. New batch of QCM sensors was washed by 96% v/v ethanol and dried, and then, 50 *µ*l of cysteamine 50 mg/ml was applied per one electrode for 5 hours. Placing the QCM sensors into wet chamber prevented from a premature desiccation during the immobilization procedure. After the incubation, the electrodes were washed by deionized water and dried. In the next step, 50 *µ*l of 5% w/w glutaraldehyde was given per one electrode, let to incubate for 5 hours, washed, and dried again. Monoclonal mouse hybridoma IgM isotype antibody against glomalin (ATCC, Manassas, VA, USA) with molecular weight 150 kDa was dissolved in PBS to concentration 1 mg/ml, 50 *µ*l of the solution was applied to interact for 12 hours in the wet box, and the sensors were washed by water and dried. In the final step, the activated iron oxide nanoparticles were shaken and 50 *µ*l of suspension containing 10 mg/ml of the nanoparticles was applied to an electrode and let to interact for 12 hours. After incubation, the electrodes were rinsed with PBS and albumin solution 10 mg/ml was injected per one electrode for one hour; then, the electrodes were rinsed with PBS, and the prepared biosensors were ready to use.

### 2.3. Sample Collection and Standard Measurement of Total Protein

Soil samples representing different habitats were selected. The samples come from the Czech Republic and Antarctica. Cadasters of villages Branisovice, Kuparovice, and Dubany represented soil type of Chernozems (high biological activity, the most fertile soil in the Czech Republic), and the cadaster of village Vlkov Cambisol soil (the most common soil in the Czech Republic). The second part of the samples represents a certain extreme (in terms of biological activity) and comes from Antarctica (James Ross Island, Deception Island and Bernardo O'Higgins Riquelme base in the Antarctic Peninsula). Soil samples were taken from a depth of 0.05 to 0.15 m in topsoil (samples from the Czech Republic) and from a depth of 0.05 to 0.10 m from the active layer (samples from Antarctica). The air-dried samples were sieved through a 2 mm sieve. These samples were taken and characterized by a standard method for the extraction and determination of easily extracted glomalin [22]. Extraction was performed with solution of 20 mmol/l sodium citrate (adjusted to pH 7.0) and extraction ratio 1 g of soil and 8 ml of extraction buffer. 20 mmol/l solution sodium citrate buffer is hereinafter referred to as extraction buffer. The suspension was added to an autoclave heated to 121°C and 1.4 kg/cm^2^ for 60 minutes. Immediately after autoclaving, the mixture was centrifuged at 3900 × g for 15 minutes. The supernatant was then isolated by micropipette and kept frozen until use in the experiments.

The extracts were scrutinized for total protein content using the standard Bradford method that was introduced in the 1970s [23]. The assay was performed using Total Protein Kit (Sigma-Aldrich) containing Coomassie Brilliant Blue *G* protein dye and standard protein for method calibration. The assay was measured spectroscopically in compliance with manual provided by the kit provider. Every sample was measured five times, and means and standard error of mean were calculated; glomalin content was expressed in mg respective *µ*g per one gram of dry soil (mg/g or *µ*g/g).

### 2.4. Measuring Samples by Biosensor

**Figure 1:**
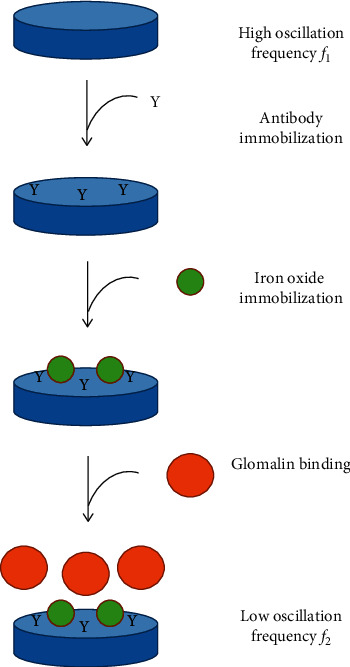
General principle of the assay.

The assay based on QCM biosensors used oscillation frequency measurement. In the beginning, the biosensors were connected to ICM Level Oscillator 10.000 MHz (ICM, Oklahoma City, OK, USA). Oscillation frequency was determined by frequency counter UZ 2400 (Grundig, Nuremberg, Germany). Initial frequency *f*_1_ was measured followed by 50 *µ*l of soil extract application, incubation of 30 minutes, washing by water, drying, and measuring oscillation frequency *f*_2_. Difference in the two frequencies Δ*f* was calculated according following formula: Δ*f* = *f*_1_–*f*_2._. Differences between signals were determined by the ANOVA test on probability levels *P* 0.05 and 0.01. General principle of the assay is depicted as Figure 1.

## 3. Results and Discussion

In the beginning of experiments, a pooled extract containing high glomalin level was chosen for the next testing of the developed method. The soil samples from cadaster Dubany municipality (Czech Republic) were chosen as the best for this purpose. The concentration of glomalin was equal to 3.47 ± 0.19 mg/g. Optimizations and calibrations of the biosensor-based assay were performed using this standard sample. Validation by testing of all the samples is mentioned in the method part.

The prepared biosensor had properly immobilized antibodies and iron oxide particles which were controlled by oscillation frequency measurement during the biosensor manufacturing. While preparation of self-assembled monolayer by cysteamine and glutaraldehyde had no effect on oscillation frequency, binding of antibodies caused decrease in the frequency equal to 753 ± 44 Hz, and binding of iron oxide nanoparticles further dropped the frequency of 958 ± 67 Hz. Blocking of surface by albumin caused drop of oscillation frequency about 132 ± 9 Hz. Repeated application of albumin had no further effect on the oscillation frequency which can be concluded by a statement that one round of surface blocking is enough to protect from nonspecific interactions with proteins.

**Figure 2:**
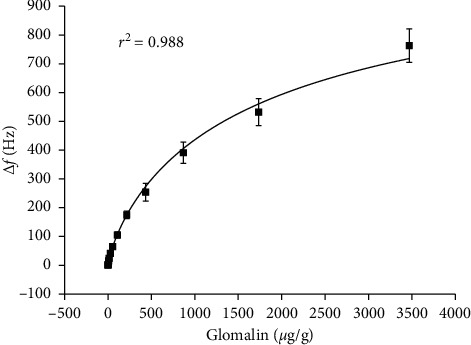
Calibration curve for glomalin using QCM biosensor. Error bars express standard deviation for five repeated measurements.

The biosensor was calibrated for the glomalin containing samples diluted up to demanded concentration using the extraction buffer. Calibration curve is depicted as Figure 2. The assay had coefficient of determination *r*^2^ equal to 0.987, limit of detection was 2.4 *µ*g/g, and the assay exerted quite good sensitivity. Though the sensitivity was slightly reduced in the range above 1 *µ*g/g, it was still usable for analytical purposes. The assay is suitable for detection of glomalin in real samples where arable field has expected median glomalin content 2 mg/g; maximal glomalin level can be found in forest ecosystem where it can reach 5 mg/g [24]. Less fertile soils have glomalin content even under 0.5 mg/g. The constructed biosensor was also compared with a biosensor where albumin 10 mg/ml was used instead of the iron oxide nanoparticles as a stabilizer. All other steps for biosensor construction were identical with the preparation of the biosensor with iron oxide nanoparticles. The biosensor with antibodies stabilized by albumin exerted a lower sensitivity and worse limit of detection (3.58 *µ*g/g) when compared to the iron oxide containing biosensor. The improved sensitivity can be attributed just to the stabilization effect of iron oxide nanoparticles that are quite dense and highly activated by carboxylic groups and thus intensively crosslinking the antibodies. Final biorecognition layer is more suitable for the detection of glomalin because of the rigidity and making the change in oscillation more corresponding to standard [25–29].

**Figure 3:**
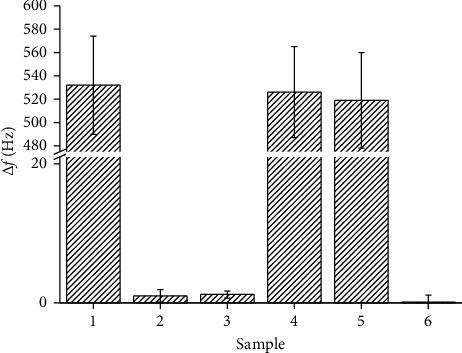
Interference testing: 1, glomalin 1.73 mg/g; 2, albumin 100 mg/ml; 3, casein 100 mg/ml; 4, glomalin 1.73 mg/g and albumin 100 mg/ml; 5, glomalin 1.73 mg/g and casein 100 mg/g; 6, extraction buffer. Error bars express standard deviation for five repeated measurements.

Interferences were estimated by analyzing albumin 100 mg/ml, casein 100 mg/ml, glomalin 1.73 mg/g (mixture of glomalin 3.47 mg/g extraction buffer 1 : 1), and mixture containing glomalin 1.73 mg/g and albumin 100 mg/ml respective casein 100 mg/ml (mixture of glomalin 3.47 mg/g and albumin respective casein 200 mg/ml). The results from the interference test are depicted as Figure 3. No statistically significant difference between results from glomalin extract sample and mixture containing glomalin extract and albumin respective casein was observed. The signal caused by albumin and casein was not significantly differing from signal of the pure extraction buffer. The interference test can be concluded by a statement that the assay is neither sensitive to interferences nor to matrix effect caused by other proteins. Albumin is a protein from blood plasma that transports nutrients, drugs, and metals [30–33]. The no interaction with the biorecognition surface on QCM is a promising result since some interactions would happen. Evidently blocking of the surface was successful resulting in biosensor surface not sensitive to unwanted interactions. The conclusion is supported by the result from casein that is a milk protein used for paint manufacturing, and it is also able to stick with various surfaces [34–36].

**Figure 4:**
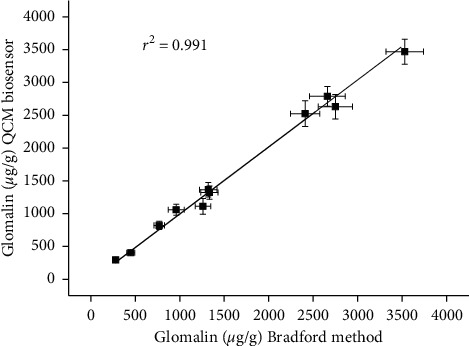
Validation of glomalin assay by biosensor to standard Bradford method. Error bars express standard deviation for five repeated measurements.

In total, eleven soil samples were analyzed by the biosensor and by the standard Bradford method. The range of glomalin content was between 279 *µ*g/g and 3.53 mg/g when the samples were analyzed by the Bradford method. The samples having glomalin content under 0.5 mg/g come from Antarctic. The results from the validation can be learned from Figure 4 where data reached by the two methods were mutually extrapolated. The extrapolation had coefficient of determination *r*^2^ equal to 0.991. This result confirms the expectation that the biosensor assay can be compared to the standard Bradford method. The achieved coefficient of determination is quite good, and it can be concluded that the biosensor assay is suitable for replacement of the standard assay. The assays were also compared for limit of detection. While biosensor had the aforementioned limit of detection 2.4 *µ*g/g, the Bradford assay exerted limit of detection 56 *µ*g/g when calibrated for the same calibration solutions.

The biosensors used in this paper were newly prepared. The sensitivity of the newly prepared biosensors was compared with the four- and eight-week old that were kept under laboratory temperature, standard humidity, and in the dark. Glomalin sample 1.73 mg/g was used for the purpose of stability studying. While the fresh biosensors provided signal 532 ± 42 Hz for the sample containing glomalin 1.73 mg/g, the four-week biosensors had signal 518 ± 39 Hz, and signal 541 ± 45 Hz was achieved when the same sample was assayed by eight-week old biosensors. It is obvious that the biosensors are long-term stable, and storage in the interval of several weeks does not cause decline of biosensors affinity toward glomalin. The biosensors used in this study were not performed repeatedly; the biosensors as well as the QCM sensors were not regenerated after the assay because it is expected that the biosensors will serve as a disposable device.

Survey of analytical parameters for glomalin assay by biosensor is given in Table 1. The assay represents an improvement to the already developed biosensor where simple immobilization antibody was chosen [37]. Comparing to the older experiment, the limit of detection was improved (the older one was 3.40 *µ*g/g), but the overall shape of calibration curve is the major advantage of the new biosensor because it has better sensitivity in the whole range of calibration. The previously published biosensor had low sensitivity for glomalin content above approximately 0.75 mg/g. The here presented biosensor does not suffer from this disadvantage which is caused by coimmobilization of iron oxide nanoparticles with recognition antibody. Both molecules were crosslinked together resulting in formation of mechanically stable layer giving better response to the analyte.

## 4. Conclusions

A biosensor for a fast assay of glomalin was prepared. The biosensor had significant advantages to the other methods: it worked in a label-free mode and was fully applicable for practical samples. The biosensor exerted promising properties due to coimmobilization of antibody specific to glomalin and iron oxide nanoparticles. The fact that no specific manipulation with samples or the assay procedure is required represents a significant advantage of the assay. The biosensor manufacturing is also simple enough to allow fabrication of large series of final products when the biosensor becomes introduced into praxis. Determination of glomalin would be also performed by the other types of immunoassays [11–16]. The here presented QCM-based biosensor appears to be easy to be introduced into praxis.

## Figures and Tables

**Table 1:** Specifications for the biosensor assay of glomalin

Specification	Value
Limit of detection	2.4 *µ*g/g
Sample volume	50 *µ*l
*r* ^2^ for calibration	0.988
*r* ^2^ for validation to Bradford assay	0.991
Interference by albumin 100 mg/ml and casein 100 mg/ml	Not significant
Type of assay/reagents needed	Label free/no applied reagents
Time per one assay	30 to 45 minutes

## Data Availability

All data used to support the findings of the study are included within the article.
